# Transcriptomic identification of IL-17/FOS-associated signaling in dartos fascia remodeling of pediatric concealed penis

**DOI:** 10.3389/fped.2026.1867230

**Published:** 2026-07-10

**Authors:** Haiyang Hu, Wenwen Liu, Zhongsong Tu, Jiao Lei, Qiao Wu, Zhangyu Yang, Xiaohou Wu, Peng Jing

**Affiliations:** 1 Department of Pediatric Surgery, Affiliated Hospital of North Sichuan Medical College, School of Clinical Medicine, North Sichuan Medical College, Nanchong, Sichuan, China; 2 Zhejiang Key Laboratory of Imaging and Interventional Medicine, Wenzhou Medical University, Lishui, China; 3 Department of Urology, Chongqing Medical University, Chongqing, China

**Keywords:** bioinformatics, concealed penis, differentially expressed genes, pediatric urology, RNA-Seq, tissue remodeling

## Abstract

Pediatric concealed penis (CP) is a congenital penile anomaly that has been associated with abnormal dartos fascia development, reduced tissue compliance, and fibrotic remodeling. While surgical correction is standard, the molecular etiology of this pathological fibrosis remains largely unexplored. This study aims to delineate the transcriptomic landscape of CP and identify candidate signaling pathways associated with dartos fascia remodeling. We performed RNA-seq on dartos fascia tissues from CP patients and phimosis control tissues, followed by bioinformatic analysis and validation using qRT-PCR, Western blot, and immunohistochemistry. RNA-seq and functional enrichment analysis identified a significantly enriched IL-17-related signaling signature in CP tissues. Experimental validation showed increased expression of key nodes such as IL-17RA, ACT1, FOS, IL-6, and PTGS2. Notably, synchronous upregulation of FOS and downstream inflammation- and remodeling-associated mediators IL-6 and PTGS2 was accompanied by fragmented elastic fibers and increased matrix deposition. These findings provide preliminary clinical tissue-based evidence that IL-17/FOS-associated inflammatory remodeling signatures are enriched in CP dartos fascia and may contribute to reduced tissue compliance and fibrosis. Further functional studies are required to determine causality.

## 1 Introduction

Concealed penis (CP) is a congenital penile anomaly that typically presents with a conical or “beak-like” appearance of the penoscrotal region; the penile shaft is normally developed but becomes buried beneath the prepubic fascia and fails to protrude normally due to abnormal attachment of the foreskin/dartos fascia (1, 2). This condition must be differentiated from obesity-related acquired buried penis and micropenis. Histopathological studies suggest that CP is associated with congenital structural abnormalities of the dartos fascia, characterized by disorganized arrangement of dartos muscle fibers rather than the normal parallel alignment (3, 4). Epidemiological data indicate that the prevalence of CP among Chinese children is approximately 0.68% (5), and among Japanese newborns it ranges from 2% to 5%, declining to 0.3% by 4–5 years of age (6). Etiological hypotheses proposed to date include poor development of the dartos leading to a deficiency of elastic fibers, inadequate fixation of the penile root skin to the pubis, and abnormalities of the suspensory ligament (7–9), among which congenital structural anomalies of the dartos fascia are considered the primary mechanism. However, the extreme scarcity of studies on the gene and protein expression profiles of CP at the molecular level represents a major bottleneck in advancing the understanding of this condition; therefore, exploring its molecular mechanisms is essential for a comprehensive elucidation of its pathogenesis (8).

The relationship between inflammation and development is bidirectional: inflammation can both worsen and ameliorate developmental outcomes. Perinatal inflammatory exposure has been independently associated with better developmental outcomes; for example, after neonatal arterial ischemic stroke, it may be accompanied by improved long-term neurodevelopment (10). However, aberrant innate immune signaling networks are thought to be associated with various neurodevelopmental disorders, and early-life inflammation can increase the risk of autism spectrum disorder, epilepsy, and other conditions (11–13). This paradoxical effect is particularly evident in the impact of fetal inflammatory response syndrome (FIRS) on lung development in preterm infants. FIRS is triggered by intrauterine infection and generally portends a poor prognosis. Its early inflammatory cytokines can promote pulmonary surfactant production and mitigate respiratory distress syndrome, yet this “pro-maturation” effect comes at the expense of alveolar architecture—inflammation directly damages alveolar walls and the microvasculature, leading to alveolar simplification and reduced alveolar numbers, which ultimately progress to bronchopulmonary dysplasia (14–16). Although the relationship between inflammation and development in concealed penis has not been extensively substantiated, it is well worth exploring.

The Interleukin-17 (IL-17) family is a class of cytokines that play critical roles in both acute and chronic inflammatory responses (17–19). Members of this family bind to their cognate IL-17 receptors, activating multiple downstream signaling pathways, including NF-κB, MAPKs, and C/EBPs. This activation induces the expression of various pro-fibrotic factors (such as IL-6 and PTGS2/COX-2), inflammatory cytokines, and chemokines, thereby promoting fibroblast activation and fibrotic processes, including aberrant collagen deposition (20). Mechanistically, the receptor-proximal adaptor protein Act1 (NF-κB activator 1) has been confirmed as the core mediator of IL-17A signal transduction (21–23). Previous studies have indicated that the role of IL-17 is becoming increasingly prominent in various inflammatory (24) and autoimmune diseases—such as psoriasis (25), rheumatoid arthritis (RA) (26), and multiple sclerosis (MS) (27)—as well as in neoplastic diseases (28). Although IL-17/FOS-associated signaling has been implicated in inflammatory and remodeling-related disorders (29–31), its expression patterns and potential regulatory role in CP remain unknown.

**Figure 1:**
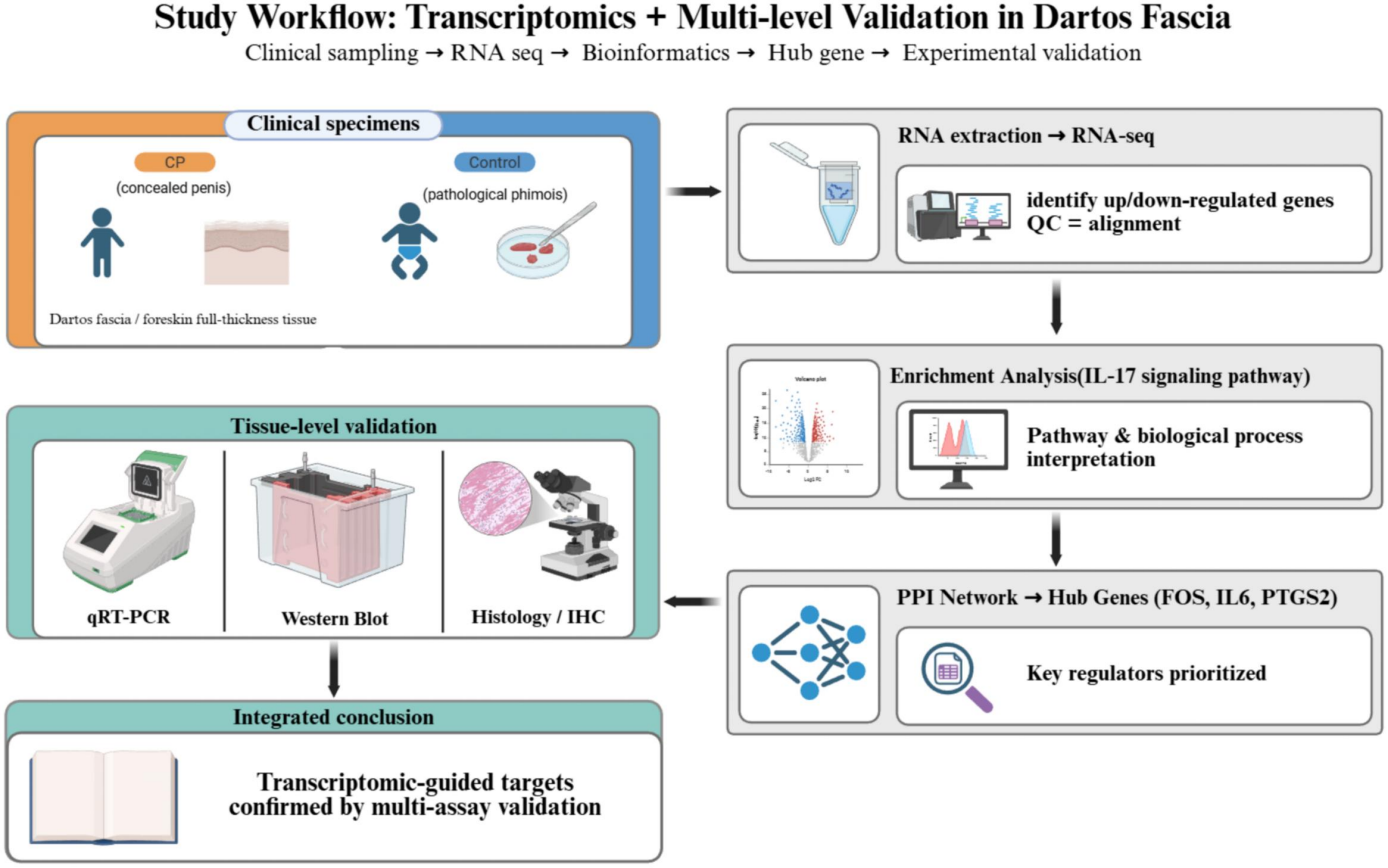
Experimental framework of the study. Integration of clinical sampling, RNA-Seq transcriptomics, and multi-level validation of the IL-17/FOS axis in an independent CP cohort.

In this study, we analyzed clinical tissue specimens obtained from children with CP and characterized their transcriptomic landscape using high-throughput RNA sequencing (RNA-seq) (32). Through integrated bioinformatic analyses, including GO enrichment, KEGG pathway analysis, and GSEA (33), we identified differential expression of FOS, PTGS2, and IL6, together with enrichment of the IL-17 signaling pathway, which may be involved in CP pathogenesis. On this basis, we performed multi-level experimental validation to assess the expression patterns of these key molecules and signaling components and to examine their association with CP pathology (23). Given the limited molecular evidence currently available in CP, this study aims to characterize transcriptomic alterations associated with CP and to propose a preliminary, data-supported model of IL-17/FOS-associated dartos fascia remodeling (9). This study was designed as a hypothesis-generating clinical tissue study rather than a functional validation study (Figure 1).

## 2 Materials and methods

### 2.1 Sample collection, ethical approval, and patient follow-up

This was an exploratory case-control clinical tissue study integrating bulk RNA-seq and molecular validation in surgically obtained dartos fascia-containing specimens. Full-thickness preputial tissue sections containing the dartos fascia were collected from pediatric patients aged under 12 years undergoing surgery at the Affiliated Hospital of North Sichuan Medical College. All tissue procurement procedures followed a standardized clinical protocol to ensure specimen integrity and consistency. Initially, samples from 5 CP patients and 5 age-matched phimosis control tissues were used for transcriptome profiling. The discovery RNA-seq cohort included 5 CP and 5 phimosis control tissues. The baseline clinical characteristics of the study participants, including group assignment, age, sex, surgical context, and specimen use (Supplementary Tables S10, S12). For molecular validation, an independent set of specimens was used for qRT-PCR, Western blotting, EVG staining, and IHC. No specimen used for RNA-seq was reused for validation. For molecular analyses, full-thickness preputial tissue sections containing the dartos fascia were collected im-mediately after surgery. For histological analyses, paraffin-embedded full-thickness pre-putial tissue sections containing the dartos fascia were used to preserve tissue architecture.

Full-thickness preputial tissue sections containing the dartos fascia were collected from the CP group during reconstructive surgery, whereas age-matched phimosis control tissues were obtained from circumcision procedures for simple phimosis. To minimize sampling bias, dartos fascia-containing tissues were dissected from comparable anatomical regions of the preputial/penile shaft tissue under the same surgical protocol. Visible epidermis and excessive subcutaneous fat were carefully removed before RNA/protein extraction. For histology, full-thickness sections were retained to preserve tissue architecture. All diagnoses were confirmed by three senior pediatric urologists. The diagnostic criteria for CP were: a short penile appearance in the natural state, with a conical or “bird-beak” foreskin, accompanied by phimosis. The key physical sign was that a normal-sized penile body could be revealed by pushing the pre-pubic fat pad posteriorly, but the penis retracted rapidly upon release of pressure. Additionally, controls with recurrent balanoposthitis, lichen sclerosus, prior penile surgery, congenital penile anomalies, systemic inflammatory disease, recent infection, or recent corticosteroid/anti-inflammatory treatment were excluded. Cases of micropenis and buried penis secondary to simple obesity were strictly excluded (9). *Ex vivo* tissues were rinsed with PBS, cut into blocks of 50–100 mg, flash-frozen in liquid nitrogen, and stored at −80 °C. Postoperative follow-up was conducted to monitor penile appearance, incision healing, and symptom improvement. The present validation study was performed in accordance with the Declaration of Helsinki and approved by the Ethics Committee of the Affiliated Hospital of North Sichuan Medical College, a teaching hospital of North Sichuan Medical College (Approval No. 2025ER420-1). Written informed consent was obtained from the legal guardians of participants included in the validation cohort. Transcriptomic profiling was used as the discovery component of this work to identify candidate pathways for subsequent validation.

### 2.2 Transcriptome sequencing and DEG analysis

A total of ten pediatric patients (five CP and five phimosis) were recruited, with diagnoses independently validated by three senior pediatric urologists based on a standardized clinical pathway. Despite the modest sample size, the samples possessed high clinical homogeneity, and strict False Discovery Rate (FDR) correction was rigorously applied to minimize false positives. Full-thickness preputial tissue sections containing the dartos fascia were collected and entrusted to OE Biotech, Inc., (Shanghai, China) for sequencing and library construction. Raw sequencing data were subjected to quality assessment and read alignment. Differential expression analysis was then carried out using DESeq2 (34), with |log2FC| > 1 and adjusted *p* < 0.05 defined as the cutoff criteria. Given the limited sample size inherent to pediatric surgical tissue availability, stringent FDR correction and consistent multi-level validation were applied to ensure robustness of findings. The resulting DEGs were subsequently used for GO annotation, KEGG pathway analysis, and GSEA. Detailed sequencing quality metrics, clean data summary, and complete DEG results are provided in the Supplementary Materials (Supplementary Tables S1, S2, S5 and Supplementary Figure S1).

### 2.3 Functional enrichment analysis of DEGs

GO functional annotation and KEGG pathway enrichment analyses of the identified DEGs were conducted using the R package “clusterProfiler” (35). This was done to systematically identify key biological processes, molecular functions, cellular components, and significantly enriched metabolic or signal transduction pathways involved. Enrichment significance was evaluated using the hypergeometric test, and *p*-values were adjusted by FDR correction. Terms with adjusted *p* < 0.05 were regarded as significantly enriched.

### 2.4 Gene set enrichment analysis (GSEA)

GSEA was performed using the GSEA software package. This approach evaluates whether predefined gene sets are preferentially enriched at either end of a ranked gene list between two biological conditions (36). In the analysis, genes were first ranked in descending order based on their differential expression levels (e.g., log₂FC) between the two groups to form an ordered gene list. Subsequently, based on pre-set gene sets (such as KEGG or GO functional sets), the enrichment was evaluated by calculating enrichment scores and performing permutation tests to determine if they were significantly enriched at the extremes of the list. The reliability of the enrichment results was assessed using FDR correction and normalized enrichment scores (NES).

### 2.5 PPI

To explore the interaction landscape of the differentially expressed genes (DEGs), protein-protein interaction (PPI) analysis was performed using the STRING database. Gene identifiers were matched to STRING entries either by direct species annotation or, when direct annotation was unavailable, by BLAST alignment of gene sequences against species-specific protein sequences in STRING with an E-value cutoff of < 1 × 10^-10. Interaction relationships corresponding to the identified DEGs were extracted, ranked by interaction score, and the top 300 interactions were retained for visualization. A three-dimensional PPI network was subsequently constructed to illustrate the major interaction architecture among DEGs.

### 2.6 RNA extraction and real-time quantitative PCR

Total RNA was isolated with the Simgen Ultra-Pure Total RNA Extraction Kit (5003050), and first-strand cDNA was generated using the All-in-one First-Strand cDNA Synthesis Kit (gDNA Purge; 7316050). Expression levels of *FOS*, *PTGS2*, and *IL6* were measured by real-time qPCR with the SYBR Green qPCR Kit (Simgen), using *GAPDH* as the internal control. Relative mRNA expression was calculated with the 2^-ΔΔCT method and normalized to GAPDH (Supplementary Table S3). An additional assessment of candidate reference-gene stability, including transcriptome-based evaluation and ACTB qRT-PCR validation (Supplementary Tables S13, S14).

### 2.7 Western blot analysis

CP tissue specimens were collected, and tissue proteins were extracted using RIPA lysis buffer. Protein concentration was determined using the BCA method (Beyotime, China). Equal amounts of protein were resolved on 10% SDS-PAGE gels and subsequently transferred to nitrocellulose membranes. Membranes were blocked with 5% non-fat milk at room temperature for 1.5 h and then incubated overnight at 4 °C with primary antibodies against IL-17RA, Act1, IL-6, c-FOS, and PTGS2, together with a manufacturer-designated AP-1 antibody (detailed antibody information is provided in Supplementary Table S4). The following day, membranes were incubated with secondary antibodies for 1 h at room temperature in the dark. Subsequently, bands were detected using a chemiluminescence imaging system, and gray values were measured using ImageJ software (NIH, Bethesda, USA). Uncropped Western blot images with molecular weight markers are provided in Supplementary (Supplementary Figure S3).

### 2.8 EVG staining and immunohistochemistry

Histopathological evaluation included EVG staining and immunohistochemical analysis. Histological analyses were performed on paraffin-embedded full-thickness preputial tissue sections containing the dartos fascia. Fresh tissue specimens were fixed in 4% paraformaldehyde, embedded in paraffin, and cut into 4-*μ*m serial sections for EVG staining and immunohistochemistry. For EVG staining, sections were stained to visualize elastic fibers and collagen. Stained sections were examined and imaged under a light microscope (MSD-1000) at 10 × and 20 × magnification. For IHC, sections underwent antigen retrieval and endogenous peroxidase blocking. They were then blocked with 5% BSA and incubated overnight at 4 °C with primary antibodies against IL-17RA, α-SMA, c-FOS, IL-6, and PTGS2. Following incubation with HRP-labeled secondary antibodies, sections were visualized using DAB and counterstained with hematoxylin. Imaging was performed under a light microscope (MSD-1000) at 15 × and 40 × magnification. For semi-quantitative analysis of immunohistochemical staining, digital images were analyzed using ImageJ software (NIH, Bethesda, MD, USA). The immunoreactivity of α-SMA, c-FOS, IL-6, IL-17RA, and PTGS2 was evaluated by measuring the integrated optical density (IOD) and the percentage of positive area (%Area) in three randomly selected, non-overlapping high-power fields from each section. For each group, *n* refers to the number of biological samples (patients), whereas multiple fields were averaged within each specimen for image quantification.

### 2.9 Desmosine ELISA

For desmosine quantification, fresh-frozen full-thickness preputial tissue specimens containing the dartos fascia were used from each sample, including five CP tissues and five phimosis control tissues. Approximately 20 mg of tissue from each specimen was weighed as the initial wet tissue weight, briefly rinsed with 1 × PBS to remove residual blood, minced into small pieces, and homogenized in 200 μL of 1 × PBS, corresponding to the tissue-to-buffer ratio recommended by the manufacturer. The homogenates were stored overnight at −20 °C, followed by two freeze–thaw cycles to disrupt cell membranes. After centrifugation at 5,000 × g for 5 min at 2–8 °C, the supernatants were collected for ELISA measurement.

Desmosine levels were quantified using a Human Desmosine (DES) ELISA Kit (CSB-E12871 h; Cusabio, China) according to the manufacturer's protocol. This assay employs a quantitative sandwich enzyme immunoassay technique, with a detection range of 0.156–10 ng/mL and a sensitivity of 0.039 ng/mL. Standards and tissue homogenate samples were assayed in duplicate. Tissue homogenate supernatants were diluted as appropriate to ensure that the measured values fell within the range of the standard curve. Briefly, 100 μL of standards or samples was added to each well and incubated for 2 h at 37 °C, followed by sequential incubation with biotin-conjugated anti-DES antibody and HRP-avidin. After substrate reaction with TMB, absorbance was measured at 450 nm. DES concentrations were obtained from the standard curve. Desmosine content was then calculated by correcting for the corresponding dilution factor and total homogenate volume, converted from ng to μg, and normalized to the initial wet tissue weight. Results were expressed as μg/g wet tissue.

### 2.10 Statistical analysis

All quantitative data are expressed as mean ± SEM. Statistical evaluation was carried out using GraphPad Prism 10 software (GraphPad Software, San Diego, CA, USA). Comparisons between two groups were performed using either the unpaired Student's t-test or the Mann–Whitney U test, depending on data distribution. A value of *p* < 0.05 was considered statistically significant.

## 3 Results

### 3.1 Transcriptomic profiling and DEG identification

**Figure 2:**
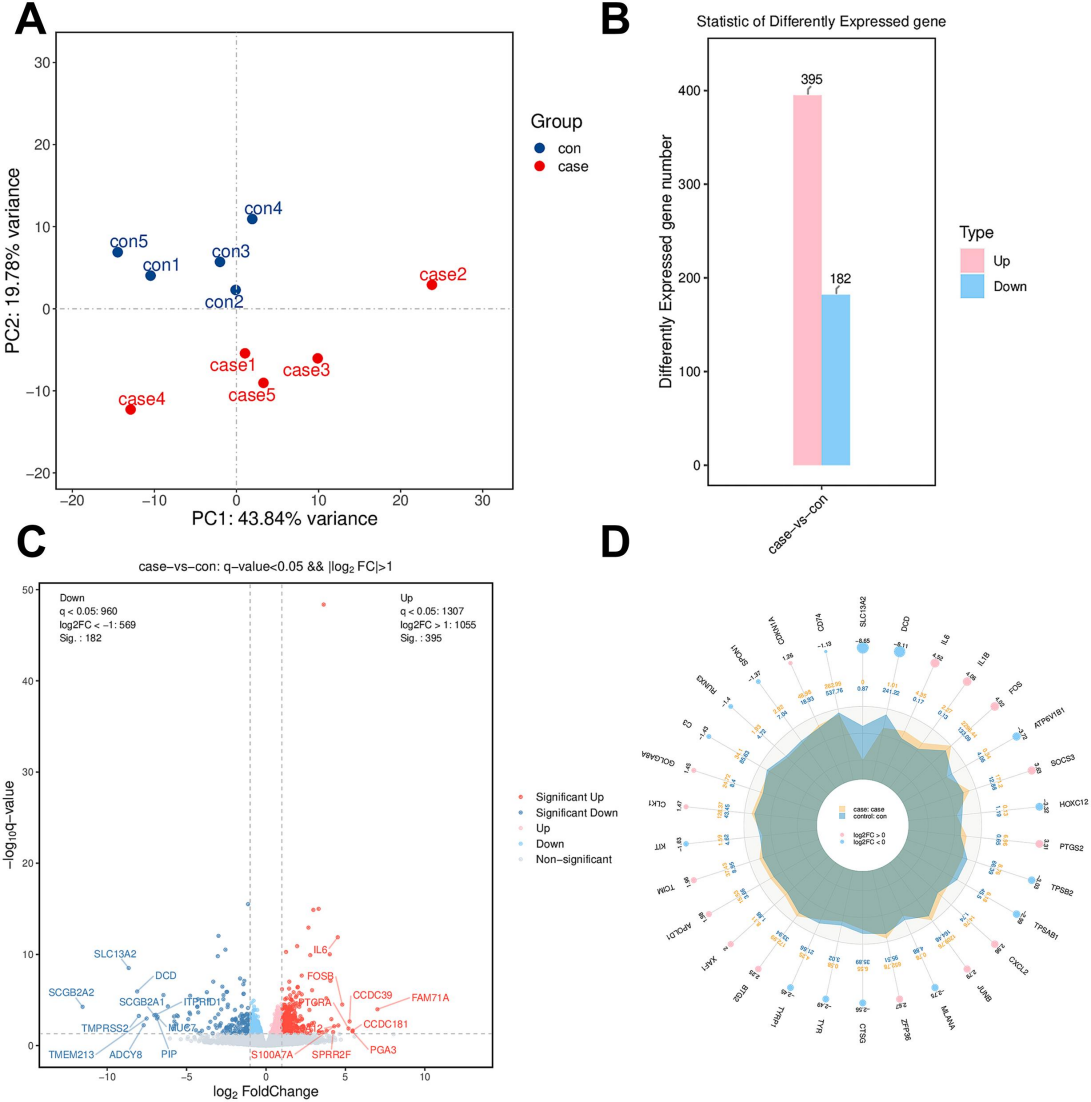
Global transcriptome profiling and differential expression analysis. **(A)** PCA score plot of CP and Control samples. **(B)** Total number of up- and downregulated DEGs. **(C)** Volcano plot of DEGs; red and blue dots represent up- and downregulated genes, respectively. **(D)** Radar chart of the top 15 upregulated and top 15 downregulated DEGs.

The clinical and specimen-use characteristics of the enrolled CP and control cohorts are summarized in Supplementary Table S10. Principal component analysis (PCA) was used to characterize the global tran-scriptomic pattern and sample distribution based on normalized RNA-seq expression da-ta. PCA showed partial separation of CP and control samples along PC2, whereas PC1 primarily reflected inter-individual variability, consistent with the heterogeneity expected in clinical tissue specimens (Figure 2A). To further evaluate whether tissue cellular composition could influence the transcriptomic comparison, a cell type marker-based composition check was performed, revealing no major differences (Supplementary Figure S4).

Subsequently, reference transcriptome sequencing was performed on 10 samples (5 in the CP experimental group and 5 in the control group), obtaining a total of 67.67 Gb of clean data (OE Biotech, Inc., Shanghai, China). Using the predefined cutoff criteria, 577 DEGs were obtained from the RNA-seq dataset (screening criteria: q-value < 0.05 and fold change > 2) (Figure 2B). Further filtering identified the top 15 upregulated and top 15 downregulated DEGs according to adjusted *p* value and absolute log2FC for visualization. Among them, FOS, IL6, and PTGS2 showed marked upregulation in CP tissues (Figures 2C,D). Similar expression trends were subse-quently observed in the validation experiments. Detailed RNA-seq quality metrics, clean data summary, and the complete list of differentially expressed genes are provided in the Supplementary Materials (Supplementary Tables S1, S2, S5 and Supplementary Figure S1).

### 3.2 Functional enrichment and pathway validation

To elucidate the biological functions driving the identified transcriptomic alterations, we performed a multi-layered enrichment analysis combining Gene Ontology (GO), KEGG pathway analysis, and Gene Set Enrichment Analysis (GSEA).

**Figure 3:**
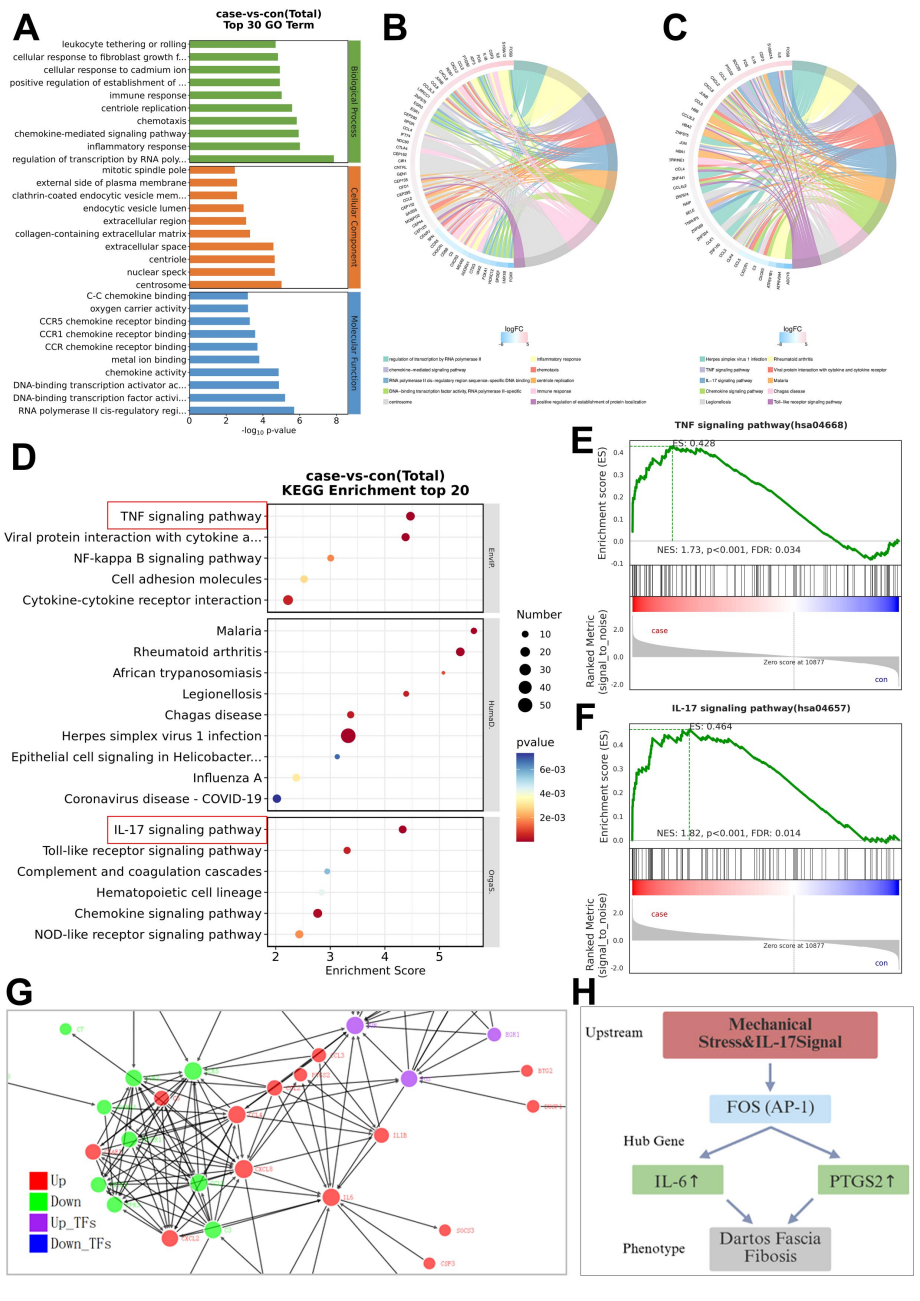
Integrated functional enrichment analysis and molecular network identification. **(A,B)** GO enrichment by biological process, cellular component, and molecular function. **(C,D)** Kyoto Encyclopedia of Genes and Genomes (KEGG) pathway enrichment analysis. **(E,F)** GSEA plots of IL-17 and TNF signaling pathways (NES > 1.5, *P* < 0.05). **(G)** PPI network identifying FOS, IL6, and PTGS2 as hub nodes. **(H)** Proposed mechanism map of the IL-17/FOS axis in CP.

First, GO functional annotation (Figures 3A,B) revealed that the DEGs were predomi-nantly enriched in biological processes critical to tissue remodeling and inflammation, in-cluding “regulation of inflammatory response,” “regulation of fibroblast proliferation,” and “immune response regulation.” These findings align with the histopathological features of CP, characterized by chronic inflammation and fibrosis. Furthermore, KEGG pathway analysis (Figures 3C,D) identified the “IL-17 signaling pathway” and the “TNF signaling pathway” as the top enriched terms. Given that TNF signaling was also enriched, we further compared IL-17, TNF, and NF-κB-related inflammatory pathways in the Supplementary Materials. These results supported prioritization of IL-17 signaling for downstream validation, while also indicating that the observed transcriptomic changes should be interpreted within a broader inflammatory network rather than as an IL-17-specific response. This finding highlighted the IL-17 signaling pathway as a prominently enriched pathway in the CP transcriptomic network.

To further verify pathway-level alterations without relying solely on differential ex-pression cutoffs, Gene Set Enrichment Analysis (GSEA) was performed. The results pro-vided quantitative evidence (Figures 3E,F) that the IL-17 signaling pathway gene set was significantly positively enriched in CP tissues (NES > 1.5, *P* < 0.05), indicating an enriched transcriptional signature rather than mere expression fluctuation. Additionally, to identify key molecular drivers within this pathway, we constructed a Protein-Protein In-teraction (PPI) network (Figure 3G). Topological analysis revealed that FOS, IL-6, and PTGS2 occupied central hub positions (Hub nodes) with the highest connectivity. Together, these data support a functionally connected relationship among these molecules and support the possibility that FOS acts as an important transcriptional regulator linked to IL-6 and PTGS2 expression in CP tissues. Based on these findings, a preliminary mechanism map was constructed to summarize the IL-17/FOS-associated signaling framework in CP (Figure 3H). The full GO and KEGG enrichment results, GSEA results for the IL-17 signaling pathway, and protein–protein interaction pairs with hub-gene ranking information are provided in the Supplementary Materials (Supplementary Tables S6–S9, S11 and Supplementary Figure S2).

### 3.3 Construction of a potential IL-17/FOS-associated signaling axis

**Figure 4:**
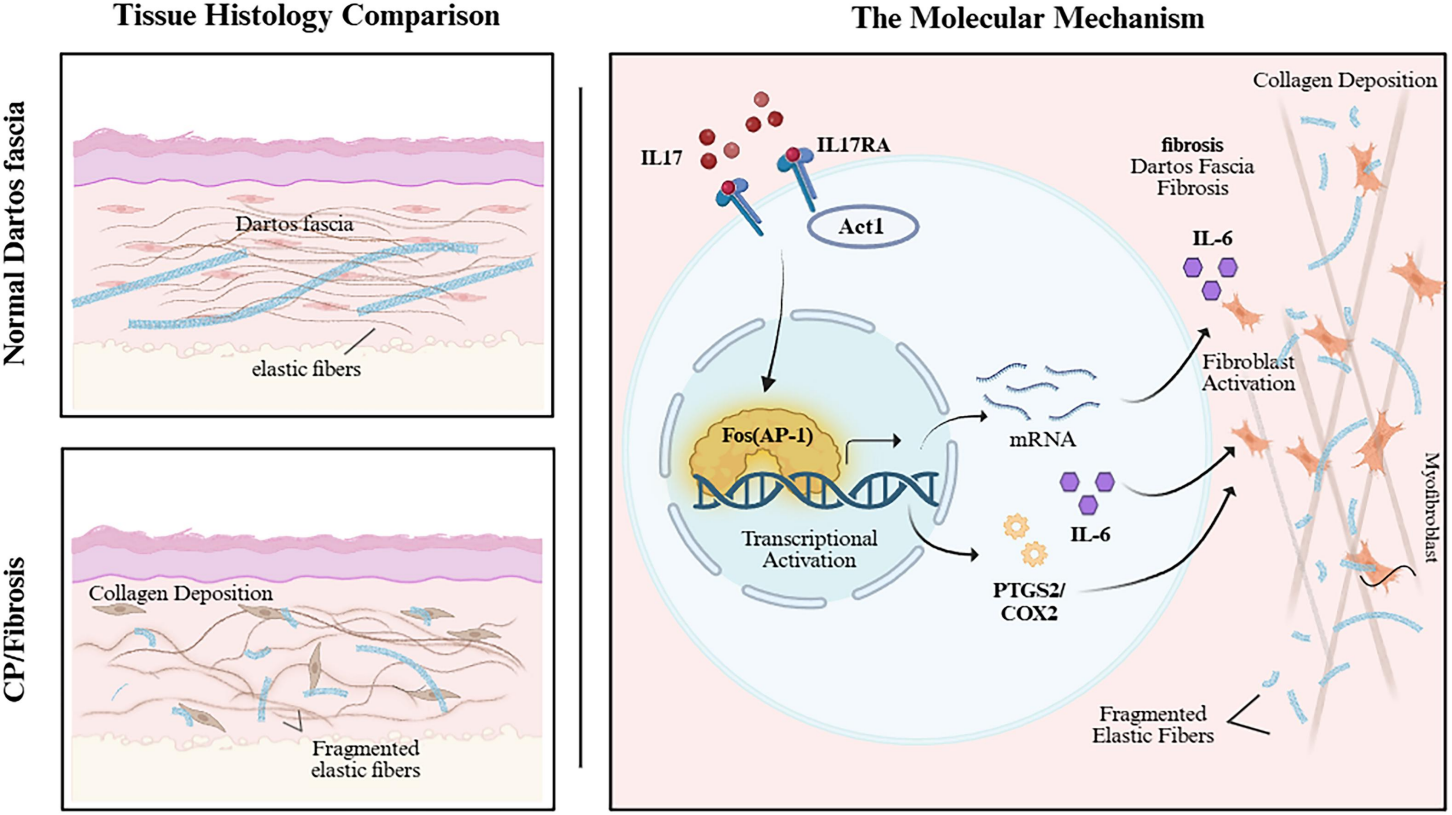
Proposed schematic model of IL-17/FOS-associated signaling in CP. IL-17 receptor-associated signaling is linked to increased FOS expression and downstream IL-6/PTGS2 upregulation, potentially contributing to fibroblast activation and elastic fiber abnormalities in the dartos fascia.

To further characterize the inflammatory-remodeling program associated with CP fibrosis, we integrated transcriptome enrichment signals, PPI network topological features, and clinical protein validation data to reconstruct a candidate IL-17/FOS-associated signaling signature (Figure 4):

GSEA enrichment analysis revealed a significant positive enrichment of the IL-17 signaling pathway gene set in CP tissues (NES > 1.5, *P* < 0.05), suggesting an IL-17 pathway-enriched transcriptional signature in CP tissues. Consistent with sequencing data, western blot analysis showed increased protein levels of IL-17RA and Act1 (37), whereas qRT-PCR analysis showed significantly increased expression of FOS, IL6, and PTGS2 in CP tissues. The increased expression of these receptor-proximal components supports the presence of IL-17-related molecular changes in CP tissues, although ligand-level activation was not directly assessed.

Within the PPI network, FOS emerged as the most prominent hub gene based on connectivity. In validation experiments, the expression level of FOS was significantly elevated. As a canonical component of AP-1-related transcriptional programs (38), the upregulation of FOS supports its potential involvement in an AP-1-associated signaling context in CP; however, the present clinical tissue data do not directly establish AP-1 complex activity or downstream target selectivity. This finding suggests that FOS may participate in linking upstream inflammatory signals to downstream transcriptional responses.

In parallel, we observed synchronous overexpression of two key effector molecules, IL6 and PTGS2, at both mRNA and protein levels. The integrated data suggest that this coordinated expression pattern may be associated with transcriptional regulation involving the FOS/AP-1 axis. High expression of IL-6 promotes fibroblast activation via paracrine or autocrine mechanisms (39), while sustained upregulation of PTGS2 (COX-2) ex-acerbates local prostaglandin metabolic disorders (40). The coordinated alteration of the “IL-17 signaling input → FOS transcriptional integration → IL-6/PTGS2 effector output” axis may help explain the phenotypes of active fibroblast proliferation, abnormal collagen deposition, and elastic fiber abnormalities observed in histopathology.

### 3.4 Experimental validation and pathological correlation

To validate the proposed mechanism, we conducted multi-level validation in clinical samples.

**Figure 5:**
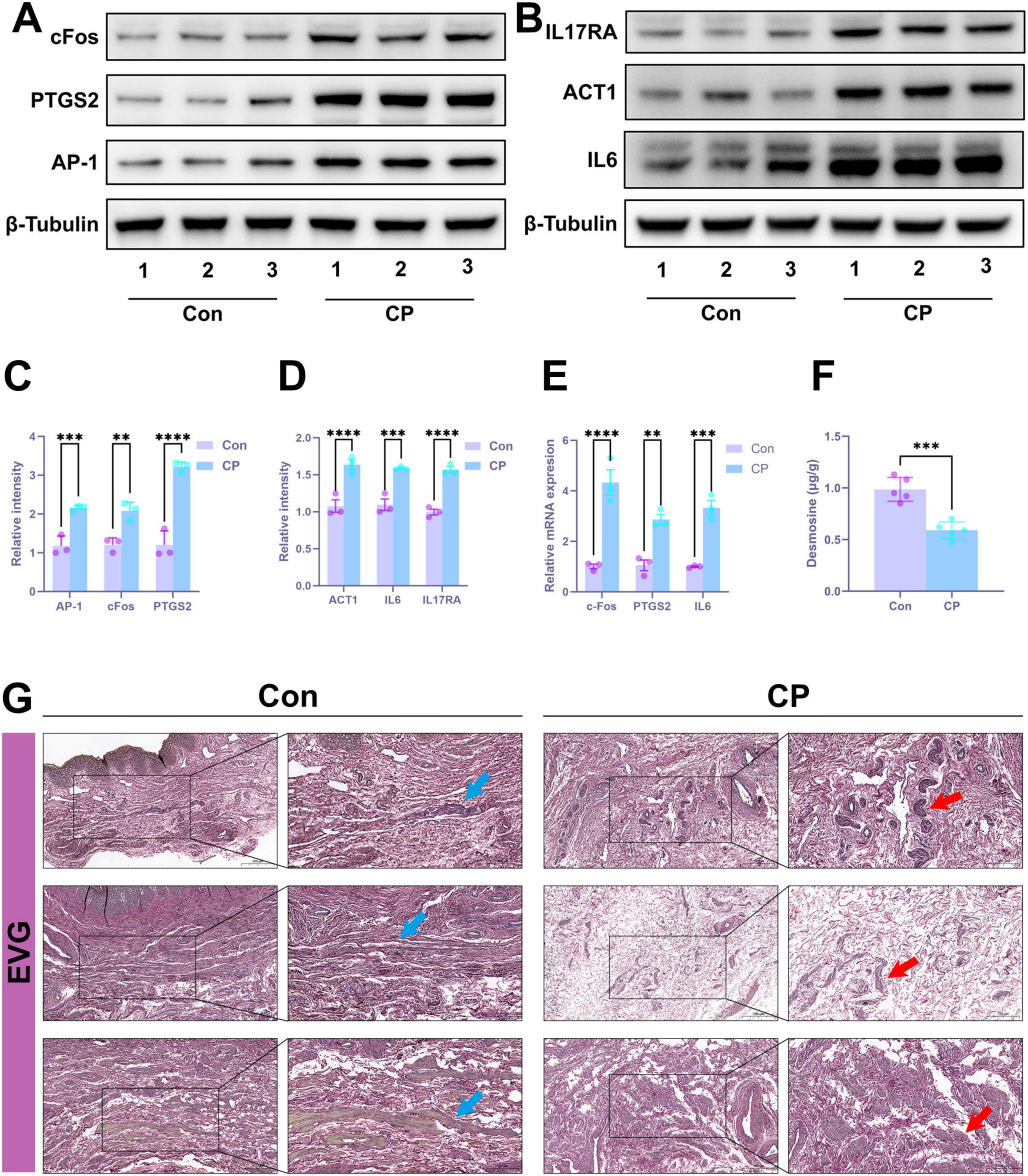
Multi-dimensional experimental validation of the signaling axis and fibrotic phenotypes. **(A–D)** Representative WB images and corresponding quantitative analysis of IL-17RA, Act1, c-FOS, IL-6, and PTGS2 protein expression, together with the signal detected by a manufacturer-designated AP-1 antibody (*n* = 3 patients/group). **(E)** Relative mRNA levels of FOS, PTGS2, and IL6 by qRT-PCR (*n* = 3 patients/group). **(F)** Quantitative assessment of desmosine levels by ELISA. Desmosine levels were significantly reduced in CP tissues compared with phimosis controls, with data presented as mean  ± SEM and each dot representing one biological sample (*n* = 5 patients/group). **(G)** Representative EVG staining from 3 patients/group. CP tissues show fragmented, granular-like elastic fibers (red arrows) within whirlpool-like collagen; Controls show thick, continuous fiber bundles (blue arrows) in a parallel arrangement. **p < 0.01, ***p < 0.001, and ****p < 0.0001 versus the control group.

Molecular Validation (WB & PCR): Western blot analysis (Figures 5A,B) served as a major validation approach, covering the upstream, midstream, and downstream targets of the signaling cascade. The results showed increased band intensities for IL-17RA and Act1, together with higher c-FOS, IL-6, and PTGS2 protein signals in CP tissues than in controls. A stronger signal detected by the manufacturer-designated AP-1 antibody was also observed; this finding should be interpreted as supportive protein-level evidence rather than direct proof of AP-1 functional activation. Quantitative grayscale analysis (Figures 5C,D) further showed that these protein-level changes were statistically significant (*P* < 0.05). To further confirm the RNA-seq findings, qRT-PCR analysis (Figure 5E) was performed on three top-ranked differentially expressed genes (DEGs) identified by our bioinformatics screening—FOS, PTGS2, and IL6. All three genes displayed significantly higher mRNA expression in CP tissues, consistent with the sequencing findings.

Desmosine ELISA-based assessment of elastin-associated remodeling: To further evaluate elastic fiber integrity at the biochemical level, desmosine, a specific cross-linking amino acid of mature elastin, was quantified using ELISA. Compared with phimosis controls, CP tissues showed significantly reduced desmosine levels (Figure 5F). This decrease paralleled the EVG-based histological findings described below and further supported reduced mature elastin-associated cross-link content and abnormal elastin-associated matrix organization within the dartos fascia of CP tissues.

Histopathological and *in situ* Expression Validation: To verify the tissue remodeling phenotype and localize the target proteins, we performed histological staining. EVG staining showed distinct histological differences between the two groups. In CP tissues (Figure 5G), the dartos fascia exhibited a disrupted parallel architecture, characterized by fragmented or granular-like elastic fibers (red arrows) embedded within dense, whirl-pool-like collagen bundles. In contrast, the control group showed a relatively normal, parallel, band-like fibrous architecture with thick and continuous elastic fiber bundles (blue arrows).

### 3.5 Quantitative analysis of immunohistochemical staining

**Figure 6:**
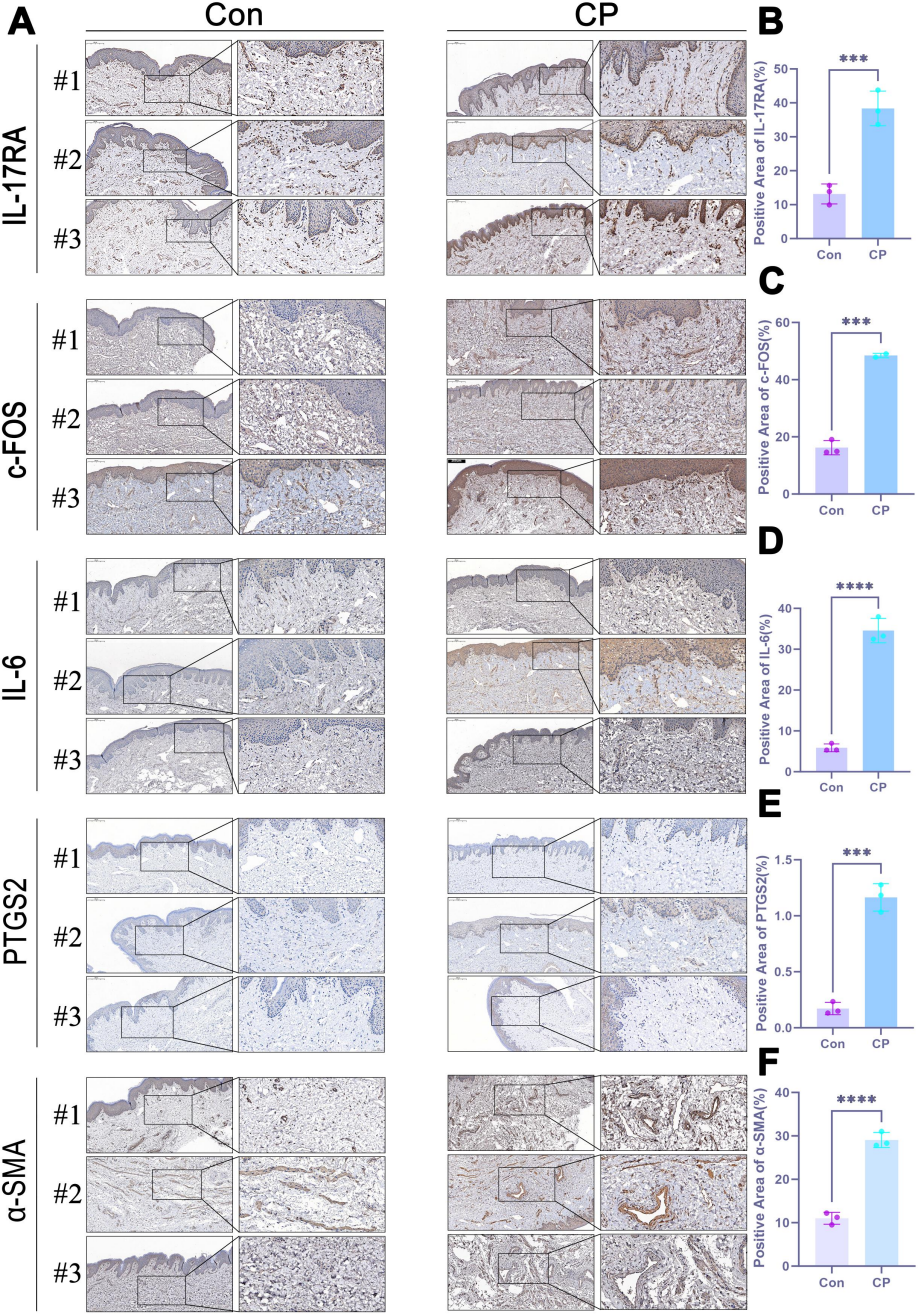
Spatial localization and semi-quantitative analysis of the IL-17/FOS axis. **(A)** Representative immunohistochemistry (IHC) images of dartos fascia (15 × and 40 ×). α-SMA confirms myofibroblast activation; c-FOS exhibits nuclear/perinuclear translocation; while IL-17RA, IL-6, and PTGS2 show robust cytoplasmic expression. **(B–F)** Semi-quantitative assessment of protein staining based on IOD and positive area (%Area), with *n* = 3 patients/group; three non-overlapping high-power fields were analyzed per section and averaged for each case. ***p < 0.001, and ****p < 0.0001 versus the control group.

To further validate the histopathological findings, semi-quantitative analysis of immunohistochemical staining was performed. As illustrated in Figure 6, CP tissues exhibited significantly increased IOD and %Area values for α-SMA, c-FOS, IL-6, IL-17RA, and PTGS2 compared with controls (*p* < 0.05). Increased α-SMA staining quantitatively supported enhanced myofibroblast activation in the fibrotic dartos fascia, whereas the elevated expression of c-FOS, IL6, IL-17RA, and PTGS2 further supported the involvement of IL-17/FOS-associated signaling. These findings were consistent with the representative IHC images and the morphological observation of stromal remodeling in CP tissues.

## 4 Discussion

### 4.1 The IL-17 signaling pathway-mediated inflammation-fibrosis cascade

Through integrated molecular analysis combined with experimental validation, this study provides initial evidence that the IL-17 signaling pathway may be involved in aberrant fibrosis in pediatric CP. Transcriptome sequencing data revealed significant enrichment of the IL-17 signaling pathway in CP tissues, with statistically significant enrichment scores. Experimental results further supported upregulation of key molecules related to this pathway. At the protein level, increased IL-17RA and Act1 expression was observed in CP tissues, while downstream mediators including FOS, IL6, and PTGS2 showed concordant changes in the validation assays at the mRNA and/or protein levels. These findings are consistent with previous reports on the role of IL-17 in fibrotic diseases (41), although the involvement of IL-17 signaling in CP has not been well characterized to date. Notably, IL-17 signaling is known to activate NF-κB and MAPK pathways through the Act1-TRAF6 axis (42), which may offer a mechanistic context for the molecular changes observed in CP and for the formation of a pro-fibrotic factor network (43). This pathway may be involved in the observed elastic fiber abnormalities and fibrosis of the dartos fascia in CP (9). From a pathophysiological perspective, our findings are compatible with a local inflammatory-remodeling microenvironment in CP tissues, although the upstream triggers and the exact sequence of signaling events remain to be determined. Persistent IL-17-related signaling may contribute to amplification of inflammatory responses and extracellular matrix remodeling, potentially promoting fibroblast activation and excessive matrix deposition (20, 21, 24, 44). These findings provide a preliminary framework for understanding the pathogenesis of CP (45). Although IL-17-associated fibrotic signaling has been described in various inflammatory and fibrotic disorders, its phenotypic consequences appear to be highly tissue-dependent (20).

Importantly, IL-17, TNF, and NF-κB signaling are biologically interconnected inflammatory pathways (46). Therefore, the enrichment of IL-17 signaling in this study should not be interpreted as definitive evidence that IL-17 is an exclusive or primary causal driver independent of TNF/NF-κB-related inflammation. Rather, based on the comparative enrichment results provided in the Supplementary Materials, together with hub-gene analysis and protein-level validation of IL-17RA, Act1, c-FOS, IL-6, and PTGS2, we describe the findings as an IL-17 pathway-enriched and IL-17/FOS-associated inflammatory-remodeling signature within a broader inflammatory network.

### 4.2 Pathological significance of FOS as a signaling hub

While the IL-17/FOS-associated signaling signature observed in this study aligns with conserved “inflammation-fibrosis” mechanisms reported in other systemic diseases, the downstream pathological consequences in CP exhibit distinct tissue-specific heterogeneity compared to classic autoimmune models.

First, regarding cellular targets and phenotypic output: In dermatological conditions like psoriasis, IL-17A predominantly targets keratinocytes, driving epidermal hyperproliferation (acanthosis) and barrier dysfunction through the STAT3/NF-κB pathway (47). In contrast, our findings in CP suggest that mesenchymal lineage cells in the dartos fascia may be important responders, rather than epithelial cells. Unlike the “hyperplastic” phenotype of psoriasis, the IL-17/FOS-associated signaling pattern in CP is linked to a “re-modeling” phenotype characterized by increased myofibroblast marker expression (α-SMA+) and stromal stiffening. This leads to a unique pathological outcome: the loss of tissue compliance rather than epithelial thickening.

Second, regarding the matrix metabolic profile: In Rheumatoid Arthritis (RA), the IL-17/FOS axis typically upregulates RANKL to promote osteoclast genesis and bone erosion (26). Conversely, in Systemic Sclerosis (SSc), the pathway focuses on excessive colla-gen cross-linking (43). Notably, the involvement of AP-1/FOS in driving collagen production has also been confirmed in keloids, hepatic fibrosis models, and more recent fibrotic studies (31, 48) indicating a shared fibrotic mechanism. Our EVG staining and molecular data reveal that CP represents a hybrid pathology comprising both “destructive” and “constructive” elements: the axis is associated with increased collagen-rich matrix remodeling and may also be related to elastic fiber fragmentation. This simultaneous matrix stiffening and loss of elasticity is functionally distinct from the joint destruction in RA, providing a mechanical explanation for the concealed or retracted clinical appearance of the penile shaft (49, 50).

Thirdly, the “Mechano-Inflammation” loop: Unlike the systemic immune dysregulation seen in SSc or RA, we speculate that abnormal local mechanical stress may interact with inflammatory signaling in CP; however, this hypothesis requires direct biomechanical and cellular validation. The dartos fascia in CP is under constant abnormal tension due to the “bird-beak” anatomical constriction. We speculate that mechanical strain may synergize with IL-17 signaling to sustain FOS nuclear accumulation (51), potentially contributing to a localized “mechano-inflammatory” cycle (41). This hypothesis is supported by parallel findings in bladder outlet obstruction (52) and tendon mechanical injury (53), where mechanical stress similarly induces inflammation and fibrosis. This highlights that while the molecular actors (IL-17/FOS) are shared across diseases (54), the triggering context in CP—developmental mechanical restraint—is unique.

### 4.3 Limitations and future perspectives

This study revealed transcriptomic and molecular alterations associated with dartos fascia fibrosis in patients with concealed penis (CP) and proposed a regulatory model centered on IL-17/FOS. However, several limitations must be acknowledged. First, this was a single-center exploratory study with a limited sample size; therefore, larger independent cohorts are needed to confirm the reproducibility of these findings. Regarding the experimental design, phimosis-derived tissue used as the control may have introduced bias due to its potential inflammatory and fibrotic background, thereby potentially underestimating expression differences between groups (55, 56). Future validation using healthy preputial tissue, where ethically feasible, is therefore warranted. Moreover, the IL-17/FOS signaling axis proposed in this study was constructed mainly on the basis of expression data and histopathological correlation, and should therefore be interpreted as a candidate mechanistic model rather than direct evidence of causality. Future studies should combine IL17A/IL17F mRNA and protein detection, immune-cell profiling, IL-17RA/Act1 inhibition, TNF/NF-κB pathway modulation, and mechanotransduction assays in CP-related models to clarify the cellular source, ligand-level activation status, and relative contributions of inflammatory and mechanical signaling to dartos fascia remodeling. Future studies should also incorporate multiple validated reference genes and blinded quantitative analyses to improve methodological rigor and reproducibility.

Despite these limitations, the consistency among transcriptomic analysis, qRT-PCR, Western blotting, histological staining, and immunohistochemistry supports the biological relevance of this model. More importantly, this study provides a preliminary molecular perspective on dartos fascia remodeling in CP research, moving beyond a purely surgical descriptive level. Future investigations should employ functional experiments and CP-related cell models to clarify whether IL-17/FOS-related signaling plays a causal role in CP-associated fibrotic remodeling. Overall, the present study provides descriptive molecular and histological evidence of CP-associated remodeling rather than mechanistic proof.

## 5 Conclusion

This exploratory transcriptomic and molecular study identified an IL-17/FOS-associated inflammatory remodeling signature in dartos fascia-containing tissues from pediatric CP. FOS, IL6, and PTGS2 were consistently upregulated, and increased expression of IL-17RA, Act1, c-FOS, IL-6, and PTGS2 was accompanied by α-SMA positivity, collagen-rich remodeling, and elastic fiber disruption. These findings provide preliminary tissue-based evidence linking inflammatory signaling signatures with dartos fascia remodeling in CP. Further studies using larger cohorts and functional cell-based models are required to determine whether IL-17/FOS-associated signaling plays a causal role in CP pathogenesis (57).

## Data Availability

The raw RNA-sequencing data presented in this study have been deposited in the Genome Sequence Archive (GSA) for Human at the National Genomics Data Center (NGDC), China National Center for Bioinformation (CNCB) / Beijing Institute of Genomics (BIG), Chinese Academy of Sciences (CAS), under accession number HRA016421 (associated with BioProject PRJCA056927). These data can be accessed at https://ngdc.cncb.ac.cn/gsa-human.

## Supplementary material

Supplementary Table S1 Complete list of differentially expressed genes identified from RNA-seq analysis.

Supplementary Table S2 Top upregulated and downregulated genes in CP tissues.

Supplementary Table S3 Primer sequences for RT-qPCR.

Supplementary Table S4 List of antibodies used for Western blot.

Supplementary Table S5 Summary of RNA-seq clean data and sequencing quality metrics.

Supplementary Table S6 Full GO enrichment results.

Supplementary Table S7 Full KEGG pathway enrichment results.

Supplementary Table S8 GSEA results for the IL-17 signaling pathway.

Supplementary Table S9 Protein–protein interaction pairs and hub-gene ranking information derived from the PPI network.

Supplementary Table S10 Clinical and specimen-use characteristics of the enrolled participants.

Supplementary Table S11 Comparative enrichment analysis of IL-17, TNF, and NF-κB signaling pathways in CP tissues.

Supplementary Table S12 Summary of biological sample sizes and quantification procedures for validation assays.

Supplementary Table S13 Transcriptome-based stability assessment of candidate reference genes.

Supplementary Table S14 Raw Ct values and stability assessment of ACTB as an additional candidate reference gene.

Supplementary Figure S1 RNA-seq quality assessment and sample-level overview.

Supplementary Figure S2 Extended enrichment plots from transcriptomic analysis.

Supplementary Figure S3 Uncropped Western blot images with molecular weight markers.

Supplementary Figure S4 Cell type marker based composition check.
