# Supplementary material for: Transcriptomic identification of IL-17/FOS-associated signaling in dartos fascia remodeling of pediatric concealed penis
Source: Front Pediatr. 2026 Jul 10;14:1867230. doi: 10.3389/fped.2026.1867230 (PMC13396118; doi:10.3389/fped.2026.1867230)
Supplement: Supplementary Table S1 — Complete list of differentially expressed genes identified from RNA-seq analysis. [file Datasheet1.zip › Supplementary File/1.Supplementary_Material.docx]

Supplementary Material

# Supplementary Data

The RNA-seq data are available in the Genome Sequence Archive for Human at China's National Genomics Data Center under accession HRA016421 (linked to BioProject PRJCA056927) at https://ngdc.cncb.ac.cn/gsa-human.

# Supplementary Figures and Tables

## Supplementary Figures


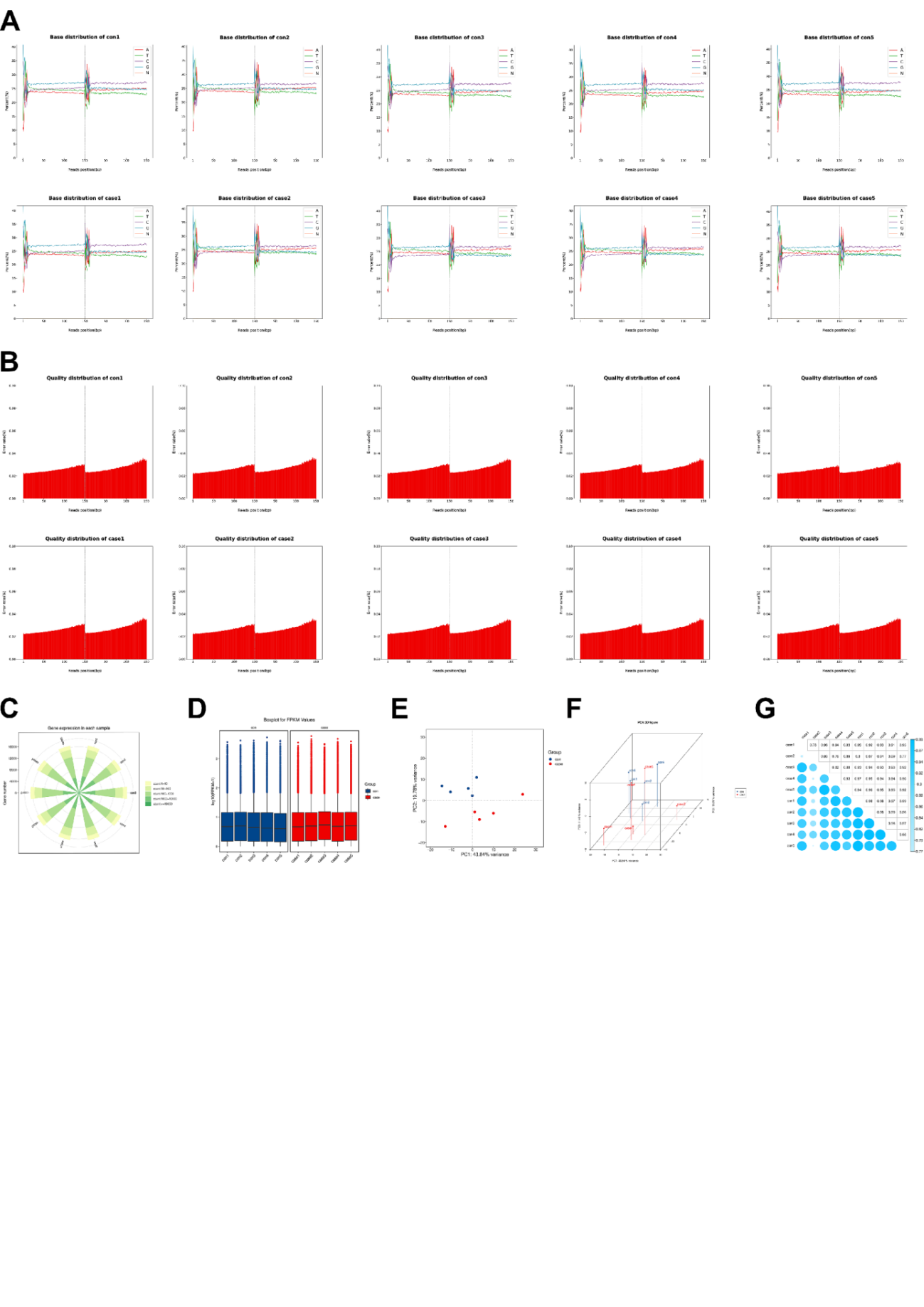


**Supplementary Figure S1. RNA-seq quality assessment and sample-level overview**. (A) Base composition across read positions for each RNA-seq sample. (B) Base quality distribution across read positions for each sample. (C) Summary of expressed gene numbers detected in each sample. (D) Boxplot of FPKM values showing the overall distribution of gene expression levels across samples. (E) Two-dimensional principal component analysis (PCA) of transcriptomic profiles. (F) Three-dimensional sample distribution plot based on multivariate transcriptomic analysis. (G) Pairwise correlation matrix among all samples. Overall, the sequencing data showed acceptable quality and consistency across samples, with relatively stable expression distributions within each group. These quality-control and sample-level analyses support the reliability of the downstream differential expression and enrichment analyses.


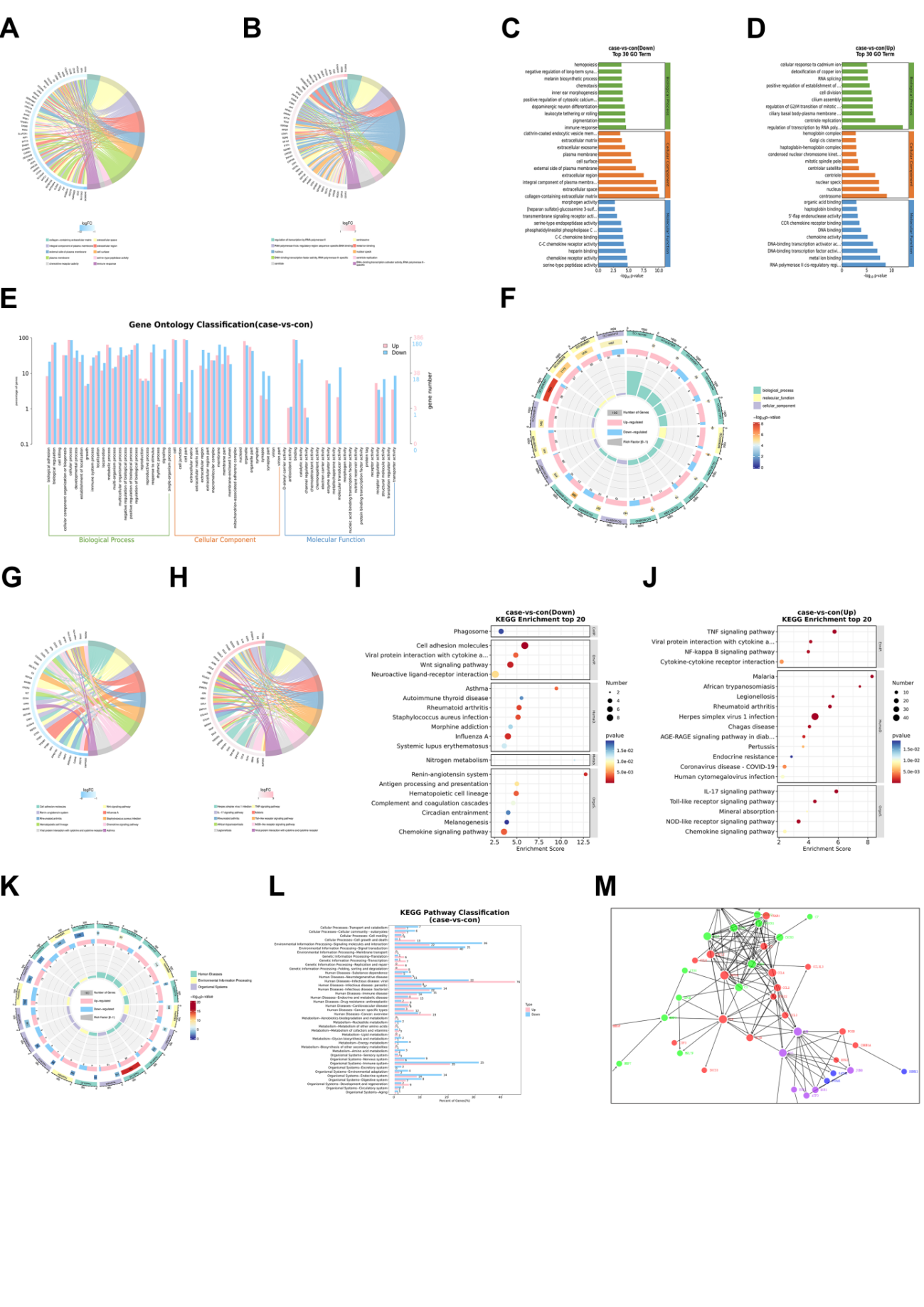


**Supplementary Figure S2. Extended enrichment plots from transcriptomic analysis**. (A-B) Chord plots of the top 10 enriched GO terms. (C-D) Bar plots of the top 30 enriched GO terms. (E) Comparison of GO level 2 functional categories between upregulated and downregulated DEGs. (F) Circos plot showing GO enrichment of differentially expressed genes (DEGs) and all detected genes. (G-H) Chord plots of the top 10 enriched KEGG pathways. (I-J) Bubble plots of the top 20 enriched KEGG pathways. (K) Circos plot showing KEGG enrichment of DEGs and all detected genes. (L) Comparison of KEGG level 2 pathway categories between upregulated and downregulated DEGs. (M) Protein-protein interaction (PPI) network.


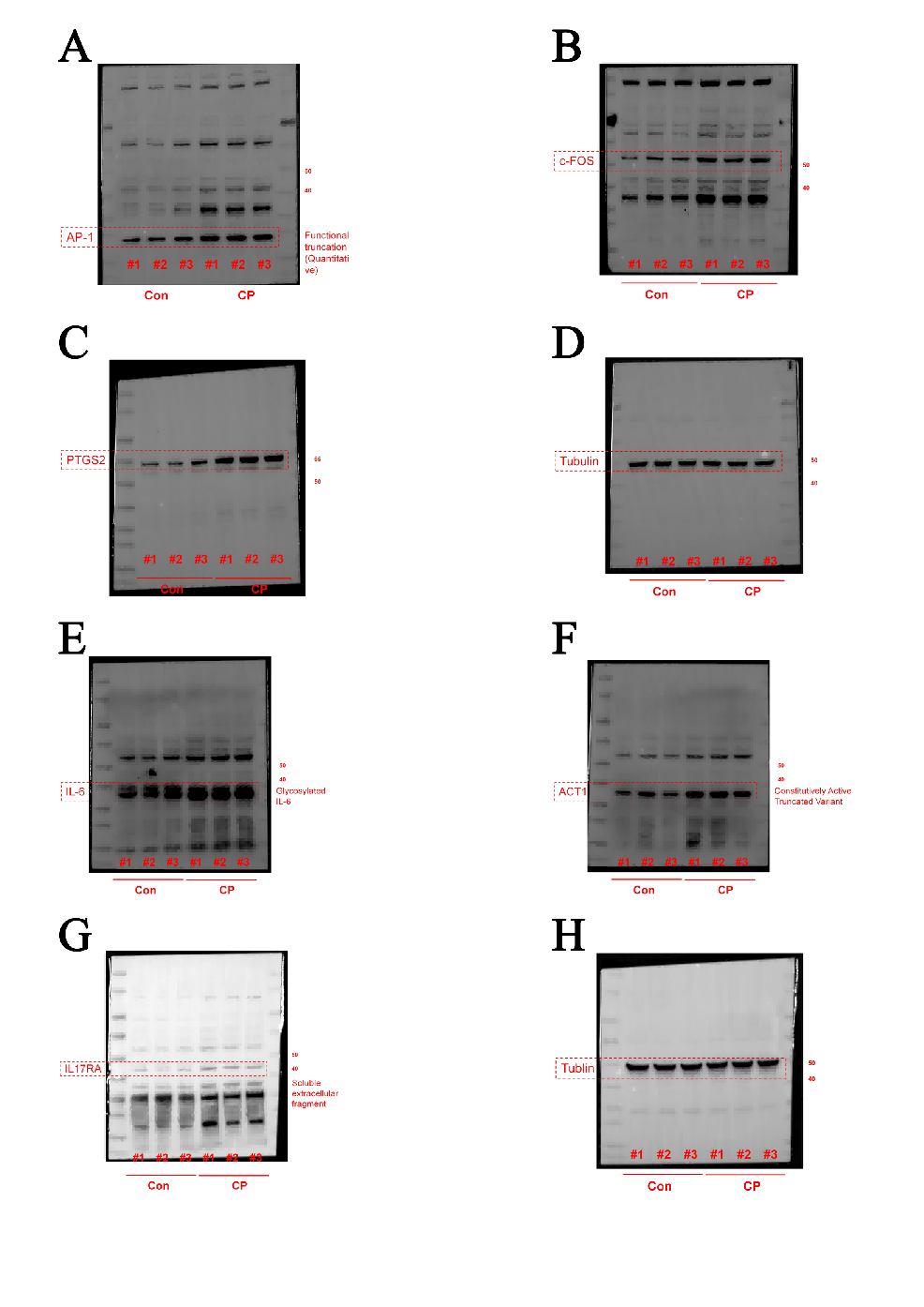


**Supplementary Figure S3. Uncropped Western blot images with molecular weight markers.** Uncropped Western blot images corresponding to the Western blot validation experiments are provided together with molecular weight markers. These images were included to improve transparency and support the reliability of the Western blot results.


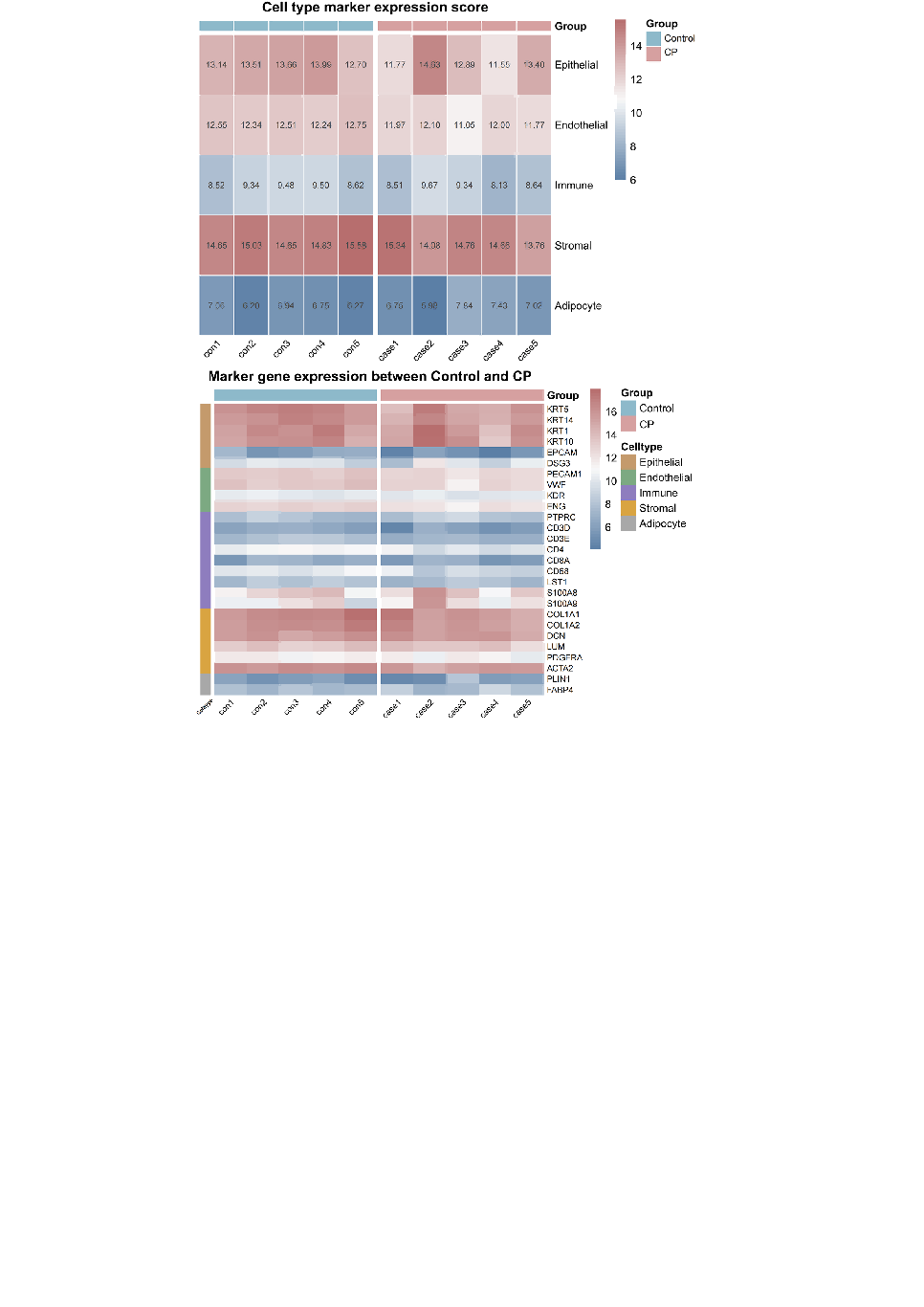


**Supplementary Figure S4. Cell type marker-based composition check.** A cell type marker-based composition check was performed to evaluate whether major differences in tissue cellular composition could influence the transcriptomic comparison between CP and control tissues. This analysis provides an additional sample-level check for interpreting the observed transcriptomic differences.

## Supplementary Tables

This document contains the legends for supplementary figures and the titles/descriptions of supplementary tables. The full supplementary tables (**Tables S1–S14**) are provided as separate files accompanying this submission.

**Supplementary Table S1. Complete list of differentially expressed genes identified from RNA-seq analysis**. Complete list of differentially expressed genes identified between CP and control tissues based on RNA-seq analysis, including gene identifiers, fold change, and statistical significance.

**Supplementary Table S2. Top upregulated and downregulated genes in CP tissues**. Top-ranked upregulated and downregulated genes identified from RNA-seq analysis in CP tissues compared with controls.

**Supplementary Table S3. Primer sequences for RT-qPCR**. Primer sequences used for RT-qPCR validation of selected genes in this study.

**Supplementary Table S4. List of antibodies used for Western blot.** Primary antibodies used for Western blot analysis, including target proteins, sources, catalog numbers, and dilution information. The anti-AP-1 antibody used in this study was designated by the manufacturer as recognizing AP-1. Detailed product information is provided in the Supplementary Materials.

**Supplementary Table S5. Summary of RNA-seq clean data and sequencing quality metrics**. Quality-filtered sequencing output for each RNA-seq sample, including clean reads, clean bases, and related sequencing quality indicators.

**Supplementary Table S6. Full GO enrichment results**. Complete Gene Ontology enrichment results for differentially expressed genes, including GO term ID, term description, gene count, enrichment significance, and adjusted p value.

**Supplementary Table S7. Full KEGG pathway enrichment results**. Complete KEGG pathway enrichment results for differentially expressed genes, including pathway name, gene count, enrichment significance, and adjusted p value.

**Supplementary Table S8. GSEA results for the IL-17 signaling pathway**. Gene Set Enrichment Analysis results for the IL-17 signaling pathway, including enrichment score-related statistics.

**Supplementary Table S9. Protein–protein interaction pairs and hub-gene ranking information derived from the PPI network**. Protein–protein interaction pairs and hub-gene ranking information derived from the PPI network analysis, including interacting genes and combined interaction scores.

**Supplementary Table S10. Clinical and specimen-use characteristics of enrolled participants**. This table summarizes the characteristics of pediatric patients whose dartos fascia-containing tissues were used for transcriptomic analysis (RNA-seq) in this study. Each row corresponds to a sample included in the RNA-seq cohort. (All data presented in this table ensure patient confidentiality).

**Supplementary Table S11.** **Comparative enrichment analysis of IL-17, TNF, and NF-κB signaling pathways in CP tissues.** This table summarizes the comparative enrichment results of IL-17, TNF, and NF-κB-related inflammatory pathways in CP tissues. These data support the interpretation that IL-17 signaling should be considered within a broader inflammatory network rather than as an isolated pathway.

**Supplementary Table S12. Summary of biological sample sizes and quantification procedures for validation assays.** This table summarizes the biological sample sizes used for RNA-seq, qRT-PCR, Western blotting, EVG staining, immunohistochemistry, and desmosine ELISA. Biological replicates refer to the number of individual patients, whereas multiple technical measurements or image fields from the same patient were averaged within each biological replicate.

**Supplementary Table S13. Transcriptome-based stability assessment of candidate reference genes.** This table presents the transcriptome-level stability assessment of commonly used candidate reference genes, including B2M, HPRT1, RPLP0, RPL13A, PPIA, YWHAZ, GAPDH, and ACTB. These data support the relative stability of housekeeping genes in the RNA-seq dataset.

**Supplementary Table S14. Raw Ct values and stability assessment of ACTB as an additional candidate reference gene.** This table provides anonymized raw Ct values and stability assessment of ACTB (β-actin) as an additional candidate reference gene for qRT-PCR validation. The Ct values were analyzed separately in the control and CP groups, and the low coefficient of variation supports the stability of ACTB expression across the tested samples.
