# Supplementary figures and images for: Transcriptomic identification of IL-17/FOS-associated signaling in dartos fascia remodeling of pediatric concealed penis
Source: Front Pediatr. 2026 Jul 10;14:1867230. doi: 10.3389/fped.2026.1867230 (PMC13396118; doi:10.3389/fped.2026.1867230)

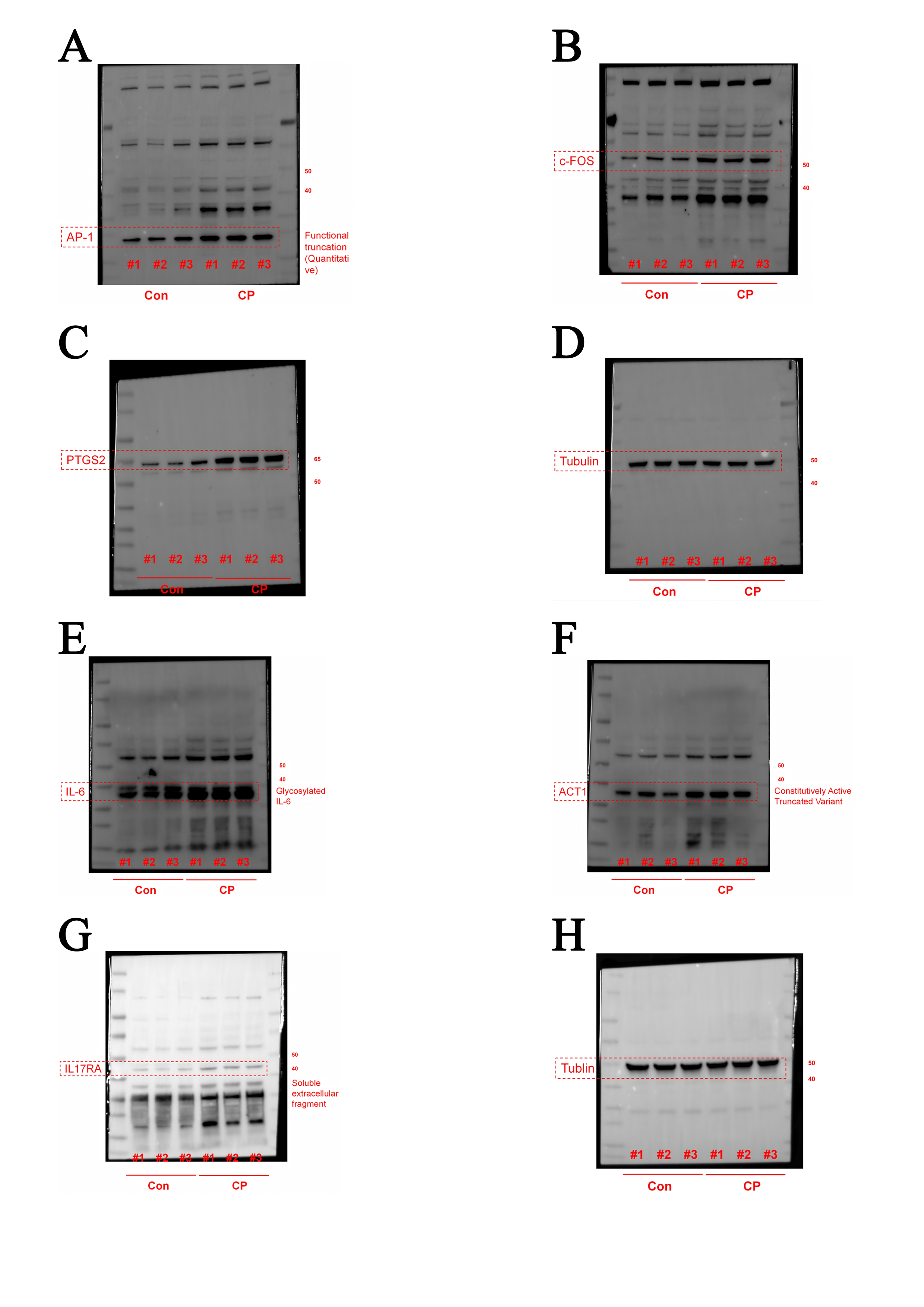

Supplement: Supplementary Table S1 — Complete list of differentially expressed genes identified from RNA-seq analysis. [file Datasheet1.zip › Supplementary File/Figure S3. Uncropped Western blot images with molecular weight markers.tif]

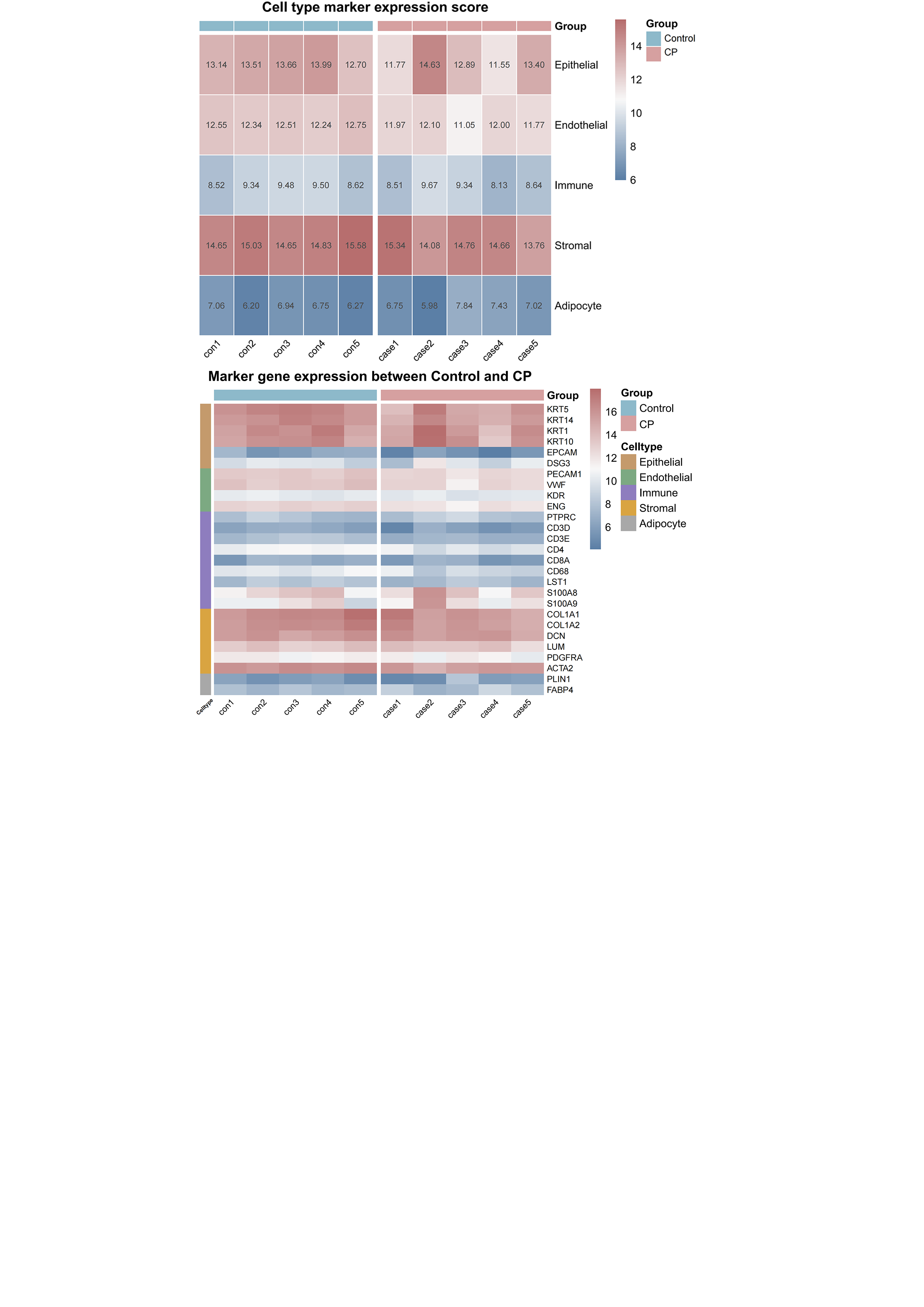

Supplement: Supplementary Table S1 — Complete list of differentially expressed genes identified from RNA-seq analysis. [file Datasheet1.zip › Supplementary File/Figure S4. Cell type marker based composition check.tif]
